# Supplementary material for: Facial Skin Microbiome Composition and Functional Shift with Aging
Source: Microorganisms. 2024 May 18;12(5):1021. doi: 10.3390/microorganisms12051021 (PMC11124346; doi:10.3390/microorganisms12051021)
Supplement: Supplementary file 1 [file microorganisms-12-01021-s001.zip › microorganisms-2873879-supplementary/supplementary Figure S1.pdf]

Figure S1. Krona plots highlighting the old and young skin samples bacterial composition. Proteobacteria composition in old cohort (a) and young cohort (b).

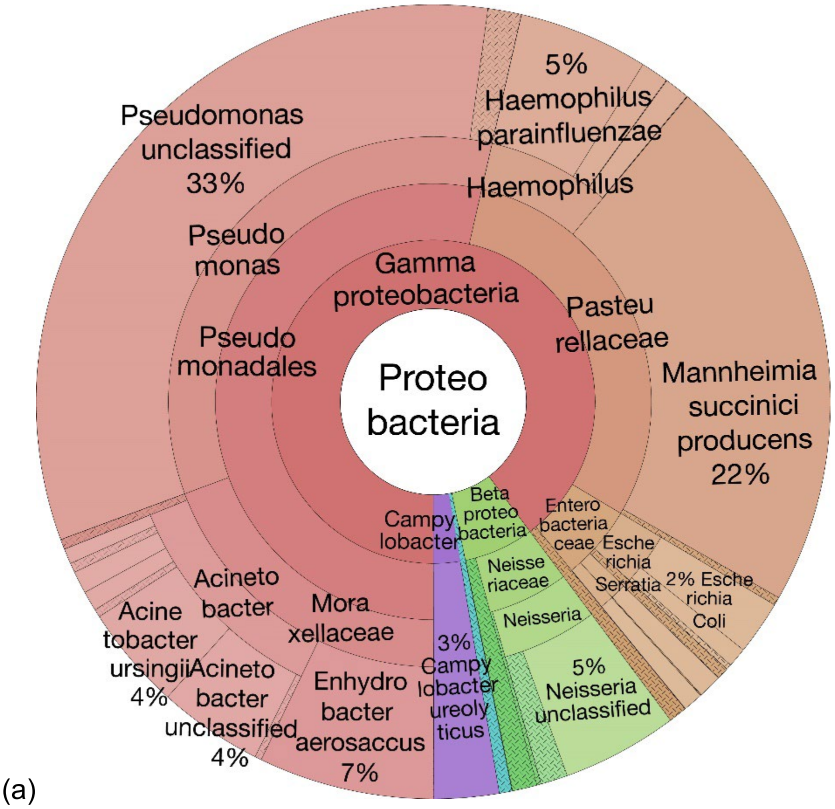

(a)

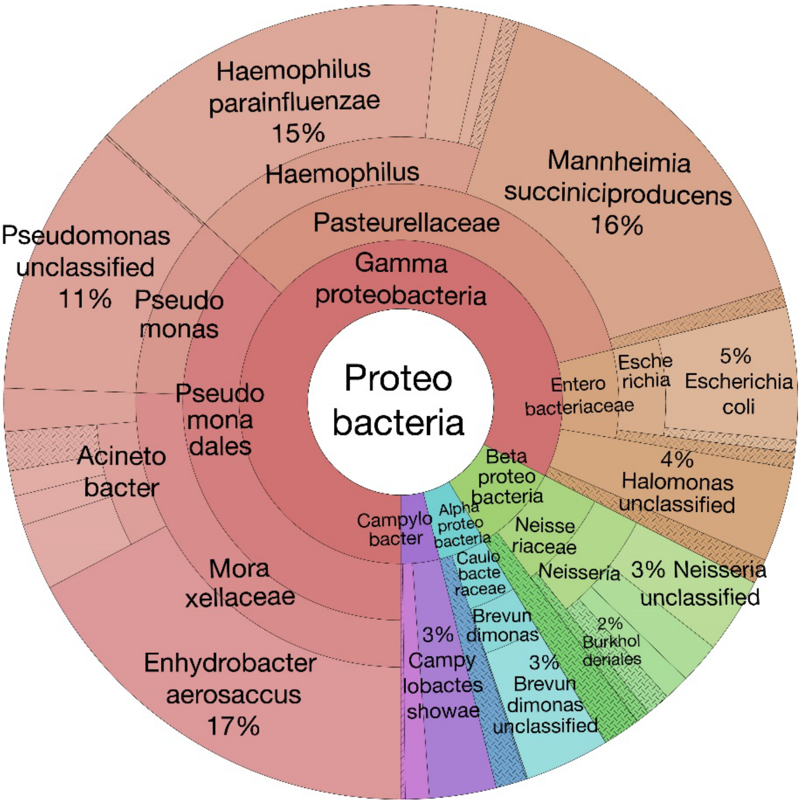

(b)
